# A novel ensemble Wasserstein GAN framework for effective anomaly detection in industrial internet of things environments

**DOI:** 10.1038/s41598-025-07533-1

**Published:** 2025-07-23

**Authors:** Rubina Riaz, Guangjie Han, Kamran Shaukat, Naimat Ullah Khan, Hongbo Zhu, Lei Wang

**Affiliations:** 1 Dalian University of Technology, Software Engineering, Dalian, 116024 China; 2 School of Internet of Things Engineering, Changzhou, 210098 China; 3 Centre for Artificial Intelligence Research and Optimisation, Design and Creative Technology Vertical, Torrens University Australia, Ultimo, NSW 2007 Australia; 4 School of Computer Science, University of Technology Sydney, Sydney, 2007 Australia; 5 School of Information Science and Engineering, Shenyang Ligong University, Shenyang, 110159 China; 6 Dalian University of Technology, Software Engineering, Dalian, 116024 China

**Keywords:** Industrial internet of things, Data augmentation, Deep learning, SMOTE, Machine learning for IIoT, Synthetic data generation, Mathematics and computing, Computational science, Computer science, Software

## Abstract

Imbalanced datasets in Industrial Internet of Things (IIoT) environments pose a serious challenge for reliable pattern classification. Critical instances of minority classes (such as anomalies or system faults) are often vastly outnumbered by routine data, making them difficult to detect. Traditional resampling and machine learning methods struggle with such skewed data, usually failing to identify these rare but significant events. To address this, we introduce a two-stage generative oversampling framework called Enhanced Optimization of Wasserstein Generative Adversarial Network (EO-WGAN). This enhanced WGAN-based Oversampling approach combines the strengths of the Synthetic Minority Oversampling Technique (SMOTE) and Wasserstein Generative Adversarial Networks (WGAN). First, SMOTE interpolates new minority-class examples to roughly balance the dataset. Next, a WGAN is trained on this augmented data to refine and generate high-fidelity minority samples that preserve the complex non-linear feature distributions characteristic of IIoT data. Unlike prior SMOTE and GAN methods, our framework leverages the Wasserstein loss for more stable training. It incorporates an optimized sampling strategy to ensure that the synthetic data meaningfully extends the classifier’s decision boundaries. Integrating an advanced oversampling technique with a critic-guided generative model significantly improves minority-class recognition, eliminating the need for extensive feature engineering or domain-specific tuning. We validate EO-WGAN on an IIoT cybersecurity dataset (UNSW-NB15) and several other imbalanced benchmarks. The proposed method consistently outperforms state-of-the-art oversampling techniques, achieving up to 95.2% accuracy (with precision and recall of 94.6% and 95.4%, respectively) in our experiments. EO-WGAN offers a scalable and cost-effective solution for anomaly detection and predictive maintenance in Industrial Internet of Things (IIoT), and its generality makes it applicable to other domains that face severe class imbalance. The results demonstrate that our approach significantly enhances the detection of minority-class events, resulting in more reliable industrial analytics and informed operational decision-making.

## Introduction

Class imbalance significantly challenges reliable pattern classification in Industrial Internet of Things (IIoT) environments, particularly hindering the detection of critical minority-class instances such as anomalies or system faults^1^. The IIoT is transforming the way industries operate and adding complexity to how data is managed and analyzed, requiring new approaches to make sense of it all^2^. Among the leading issues related to this, a great example is a class imbalance, where crucial but rare phenomena (such as equipment failure) are poorly reflected. This imbalance can severely impact the performance of the machine learning model with high false negative ratios^3^. In the realm of IIoT, this would translate into missed alerts for preemptive maintenance or, even worse, unnoticed downtimes. It has become essential to innovate techniques that are intelligent enough to have representations of minorities, but do not compromise the integrity of the data^4^. The complexity of IIoT data, with its high-dimensionality and often temporal characteristics, does not lend itself to a facile and effective pattern classification^5^. To complicate these, class imbalance occurs when the crucial patterns representing anomalies or failures are much smaller than those representing normal operations. In the context of IIoT, these instances often correspond to anomalies or system inefficiencies that, if undetected, can lead to substantial operational disruptions or failures. The IIoT data, which is typically handled by traditional machine learning algorithms with perfectly balanced datasets, often experiences delays due to skewed distributions. They give an intrinsic focus on overall accuracy, bypassing the minority-class^6^. Standard approaches, which resample the minority class or synthesize samples, are still the most popular but inappropriate for capturing complex, high-dimensional interactions typical in IIoT environments^7^. Even these methods risk overflowing the minority-class by adding redundant information interactions or capturing all those nuances only characterizing the majority class.

Recent approaches to GANs offer a novel path through this challenge, yielding promising results because they can learn and mimic the statistical properties of the input data distribution. Specifically, for WGAN, this means that synthetic data from WGANs should not be biased by particular aspects of the most critical issues with traditional resampling methods^8,9^. On the other hand, WGAN, which utilizes the Wasserstein distance, offers a powerful solution to various training problems and mode collapse issues that persist in standard GANs. In general, deploying a WGAN in the IIoT environment is not trivial. The multi-modality of IIoT data in dealing with the importance of temporal dynamics and the prevalence of rare but critical events calls for an adapted approach to exploit the potential of WGANs fully^6^. Such IIoT-specific challenges are approached using EO-WGAN to synthesising minority-class data control, boosting the training set without losing valuable information or containing remarkable noise. Generate realistic and high-quality synthetic samples to create a more balanced dataset, thereby enhancing the classification model’s performance within the IIoT framework. According to the most recent literature, EO-WGAN can be traced back to its roots, primarily focusing on the foundational work discussed by Sharma^10^. Ding et al.^11^ further expanded the base work and integrated GANs with the ensemble to find solutions to the issue of class imbalance, resulting in a holistic framework showing excellent improvements in classification performance on several real-world datasets. Yet, they face persistent challenges, including overfitting, mode collapse, and an inability to fully capture complex, high-dimensional data distributions typical of IIoT systems.

**Fig. 1:**
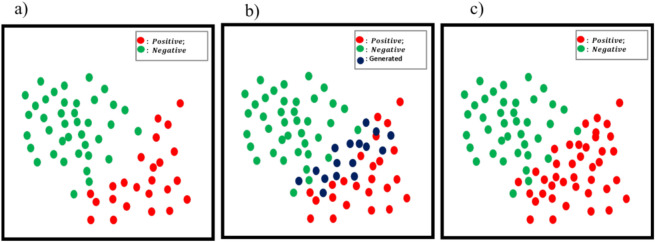
Balanced data distribution: **a**) Imbalanced data, **b**) Generated samples, **c**) Balanced data.

The novel framework overcomes the limitations of traditional methods through a two-stage approach. First, SMOTE generates synthetic minority-class samples, addressing class imbalance. Next, WGAN refines these samples, preserving realistic data distributions and avoiding noise or redundancy. This dual-stage process enhances the balance of data and ensures that the synthetic data effectively captures the complexities of real-world distributions. As shown in Fig. 1, EO-WGAN facilitates classifier differentiation between normal and anomalous patterns more effectively.

The proposed framework enhances IIoT systems by improving prediction accuracy, resulting in more proactive and informed operational strategies. Empirical results demonstrate that EO-WGAN outperforms conventional GANs and state-of-the-art resampling methods, significantly increasing metrics such as F1 score, recall, accuracy, and precision. These improvements increase predictive capacity, improving operational efficiency and reliability in industrial applications. Our approach addresses the gap in the existing literature on using WGAN for imbalanced data classification in IIoT, providing valuable information for future research and practical applications^12^. Our primary contribution lies in the innovative integration and optimization of SMOTE and WGAN to create the EO-WGAN framework. This approach is not merely a combination of existing methods but involves significant enhancements tailored to the specific challenges of IIoT data, characterized by high dimensionality, noise, and class imbalance. The acronyms used in this paper are listed in Table 1.

This paper’s key contributions include:

1. Proposing EO-WGAN, a novel two-stage oversampling framework combining SMOTE with optimized WGAN training to manage class imbalance in complex IIoT data effectively.
2. Demonstrating significant improvements in performance metrics (accuracy, precision, recall, and AUC) across multiple benchmark IIoT datasets, validating the general effectiveness and applicability of the proposed method.
3. Developing a critic guided optimization strategy within WGAN training, explicitly designed to capture complex, nonlinear distributions of minority-class samples, significantly enhancing synthetic data realism and improving accuracy in anomaly detection and predictive maintenance scenarios.
4. Introducing an adaptive hyperparameter tuning approach within the EO-WGAN framework, enabling efficient model convergence and significantly reducing training instability typically associated with traditional GAN-based methods.

The paper is organized as follows. Section 2 reviews related work to position our study within the broader context of imbalanced data classification. Section 3 introduces the proposed methodology, followed by experimental evaluation and analysis of the EO-WGAN framework in Section 4. Finally, Section 5 concludes the study with a summary of key findings.

**Table 1:** List of acronyms.

Abbreviation	Description
IIoT	Industrial Internet of Things
EO-WGAN	Enhanced Optimization WGAN
SMOTE	Synthetic Minority Oversampling
FPR	False Positive Rate
GAN	Generative Adversarial Network
PCA	Principal Component Analysis
FNR	False Negative Rate
RF	Random Forest
SVM	Support Vector Machine
API	Application Programming Interface
G-Mean	Geometric Mean
ML	Machine Learning
NN	Neural Network
CNN	Convolutional Neural Network
LSTM	Long Short-Term Memory
DT	Decision Tree
TL	Transfer Learning
RGB	Red Green Blue
F1	F1 score

## Related work

While traditional resampling techniques, including SMOTE and its variants, partially address class imbalance, their effectiveness diminishes with complex, high-dimensional IIoT data. GAN-based methods, particularly WGAN, offer improved stability and realism in synthetic data generation. However, existing approaches have limitations in directly tackling IIoT-specific challenges, motivating our refinement through EO-WGAN. This section will explore how more advanced techniques, including GAN-based frameworks, are now being integrated to address these limitations and improve classification performance in such environments.

### Traditional methods

Several methods have been proposed to address class imbalance, but they often fail in complex IIoT environments. Building on these existing methods, we now present the detailed methodology of the EO-WGAN framework. This section highlights literature on strategies adopted for tackling class imbalance, ranging from traditional resampling methods, such as SMOTE, to state-of-the-art and more sophisticated techniques that involve generative models. WGANs provide better training stability and sample quality; however, most architectures built in this approach are not able to fully adhere to the very stringent conditions of IIoT environments, including the high precision required in the case of predictive maintenance^13^. Our proposed novel EO-WGAN method builds upon these foundations. It introduces enhancements that can be applied to their baseline model to meet the nuanced requirements of IIoT applications and address the existing critical gap. GANs, introduced by Goodfellow et al.^8^, have significantly impacted the domain of machine learning. Anomaly detection plays a crucial role in identifying rare or unusual patterns that deviate from expected system behavior, particularly in safety-critical domains such as Industrial IoT^14,15^. Traditional approaches often rely on statistical thresholds, clustering, or supervised learning, but these techniques typically struggle with imbalanced data and high-dimensional feature spaces. Recent advancements leverage deep generative models, including autoencoders and GANs, to better capture complex data distributions and detect subtle anomalies^16^. However, these methods often face limitations in training stability, mode coverage, and sensitivity to noisy minority-class samples.

### Generative models

In GANs, two neural networks, the generator and the discriminator, participate in a competitive training process. The generator aims to create data that mimics the real data, while the discriminator evaluates the authenticity of the data, determining whether it is real or synthetic^17^. When trained to convergence, the adversarial training process seems promising for generating high-quality data. It thus opens the door to a new horizon in data augmentation and synthetic sample generation for imbalanced datasets^18^. GANs can produce very close-to-real synthetic samples; hence, they have been widely used in cases with a class imbalance in classification tasks. Thus, applying GANs to minority-class data helps improve model performance by balancing datasets during classification, as some studies have demonstrated in the domain of GAN application^19^. In some studies, this application has been explored, and they have shown that it enables an effective increase in the classifier’s sensitivity towards minority classes, resulting in improved classification accuracy for minority classes in imbalanced settings. As successful as GANs have been, they have faced difficulties with mode collapse and training instability. All this provoked the advent of WGAN by Arjovsky et al.^9^.

WGANs introduced the idea of the Wasserstein distance as a loss function for GAN training that is more numerically stable and semantically meaningful. Thus, WGANs appear to be even more suitable for the class imbalance problem due to the higher quality and diversity of the minority samples and the advanced state of the application of GANs in dealing with class imbalance, especially in the most complex classification tasks stated by Bhatia et al.^20^. The research carried out by Gupta et al.^21^ employs strong methodologies to represent high-quality synthetic data through the combination of improved optimization techniques and adaptive learning rates, thereby enhancing the performance model of pattern classification. Some other simplistic techniques stated by Elreedy et al.^22^ include the random oversampling of instances of the minority-class, randomly sampling the majority class at a ratio of two to one with the minority-class, and random undersampling or removal of majority-class. More sophisticated methods, such as SMOTE and its derivations (Borderline-SMOTE, SVMSMOTE, and ADASYN), aim to create new synthetic instances for the minority-class^13^.

### Challenges

Most of these methods do not consider the underlying distribution of classes with minimal cases, leading to over-generalization or noise and detecting anomalies^23,24^. Researchers have been synthesizing new instances with GANs to better represent the characteristics of the minority class or in conjunction with resampling methods^25^. Random oversampling (ROS) and random undersampling (RUS) approaches are frequently used to address class imbalance^26^. Naturally, Kuang et al.^27^ initially proposed using ROS and RUS to address the issue of class imbalance in intrusion detection. SMOTE has a relatively extensive family tree, with a collection of alternatives that have made trends in various fields, including bioinformatics, video surveillance, and anomaly detection, and have also expanded into the development area. Regular SMOTE is the foundational version, designed to generate synthetic samples in the feature space to balance class distributions. Each of these variants offers a unique approach to handling imbalanced datasets, highlighting the versatility and adaptability of SMOTE in addressing complex data challenges across various domains^28^. On the other hand, though finding some promise, this might not scale correctly in the high-dimensionality of multi-modal data in an IIoT environment. Some of the popular ensemble learning methods include Bagging and Boosting^29^. GANs for classifying imbalanced data have aroused increasing interest in their application to IIoT^30^. Compared with the consistency and better training performance of traditional GANs, attention has been paid to the WGAN variant in recent years.

Although various methods have addressed the challenge of anomaly detection^31,32^, and^33^, handling imbalanced datasets in IIoT environments, traditional oversampling techniques like SMOTE generate synthetic samples by interpolating between minority-class instances. This helps mitigate class imbalance but often leads to overfitting and fails to capture the true data distribution, especially in high-dimensional spaces. GAN-based techniques offer advantages in generating realistic synthetic samples by learning the data distribution through adversarial training. However, GANs suffer from issues like mode collapse and training instability. In contrast, WGAN, although it addresses these issues by using the Wasserstein distance as the loss function, does not explicitly tackle the class imbalance problem prevalent in IIoT environments. Based on these developments, EO-WGAN, capable of working within the IIoT data challenge framework, will further study the justification, development, and potential impacts of this new approach. Our proposed EO-WGAN model combines SMOTE and WGAN to address the class imbalance in IIoT datasets by generating high-quality synthetic samples. Using SMOTE for initial oversampling and WGAN for refining the samples, EO-WGAN generates diverse minority-class data, thereby reducing overfitting and enhancing classification performance. The method introduces an Optimized WGAN training mechanism tailored for high-dimensional multimodal IIoT data, resolving challenges like mode collapse and training instability. Our experiments demonstrate significant improvements in accuracy, precision, recall, and F1 score across various IIoT datasets, underscoring the effectiveness of this approach in anomaly detection and predictive maintenance. By overcoming limitations in traditional methods, EO-WGAN provides a robust solution to data imbalance, preserving complex data distributions and improving operational efficiency in IIoT environments, making it a valuable tool for industrial applications.

## Proposed framework

EO-WGAN employs a consistent approach using SMOTE for preliminary synthetic sample generation, followed by a refined synthetic sample creation using a WGAN. The WGAN’s critic (C) consistently guides training through the Wasserstein loss, enhancing the quality of synthetic data and classifier performance. The entire methodology, including algorithmic details, clearly delineates the roles of each component.

**Fig. 2:**
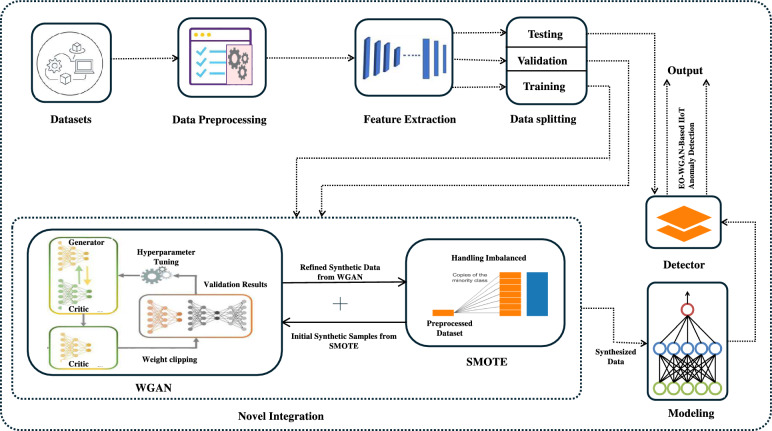
EO-WGAN Framework overview, depicting dataset splitting, synthetic oversampling via SMOTE, refinement with WGAN, and final classifier training.

The framework illustrated in Fig. 2 presents the entire pipeline of the EO-WGAN framework, showing how the dataset is split into training and test sets, the application of SMOTE for initial oversampling, the refinement of synthetic samples via WGAN, and the final classifier training to detect anomalies or faults in IIoT data. In EO-WGAN, we integrate SMOTE with Wasserstein GAN to handle severe class imbalance and generate high-quality synthetic minority-class samples. SMOTE enables the generation of interpolated samples from the minority class before entering the generative stage, which helps stabilize early training by providing WGAN with a more balanced input distribution. Unlike standard GANs, which optimize the Jensen–Shannon divergence and often suffer from mode collapse and gradient instability, WGAN minimizes the Earth-Mover (Wasserstein) distance between real and generated data distributions. This leads to smoother gradients, more stable training, and improved sample diversity, critical for generating realistic minority-class instances that enhance classifier generalization. We also selected against conditional GANs (cGANs) since they typically require well-labeled data and explicit conditioning information, which can be unreliable or missing in industrial datasets. The integration of SMOTE and WGAN provides a reliable two-stage augmentation strategy that works effectively across different levels of imbalance, without requiring architectural complexity or label dependency. This decision was further supported by recent studies^34^, which show WGAN’s improved performance in similar class-imbalanced learning problems.

### Datasets

In this subsection, we briefly describe the datasets used in our experiments discussed in the Table 2, including their characteristics and how they were preprocessed for evaluation. We also outline the experimental setup, including the hardware and software configurations used for training and assessment. Our study evaluates EO-WGAN on a diverse set of datasets, sourced primarily from the UCI Machine Learning Repository^35^, each exhibiting significant class imbalance. These datasets encompass a range of instances, from small to large-scale, where minority-class instances represent critical anomalies or failures. For example, Ecoli (335 instances, 7 features) has 5.97% minority representation for protein localization classification, while Ionosphere (351 instances, 34 features) has 35.71% minority data for radar signal classification. Similarly, Pageblocks (471 instances, 10 features) target document layout classification with a 5.94% minority-class, and Yeast (513 instances, 8 features) predicts protein cellular locations with a 9.94% imbalance. Other datasets include Wine (655 instances, 11 features, 2.74% minority), Poker (1476 instances, 10 features, 1.15% minority), Abalone (4177 instances, 8 features, 20.1% minority), and Spambase (4601 instances, 57 features, 39.39% minority). The Shuttle dataset is highly imbalanced, containing 58,000 instances with only 0.294% belonging to the minority-class.

**Table 2:** Dataset statistics and references.

Dataset	Instances (Classes)	References
UNSW-NB15	2,540,044 (10 classes, Normal: 2,218,761, Attack: 321,283)	^36^
Ecoli	336 (8 classes, Class1: 143, Class2: 77, Class3: 52, others)	^37^
Ionosphere	351 (2 classes, Yes: 225, No: 126)	^38^
Abalone	4,177 (29 classes, Class1: 391, Class2: 273, others)	^39^
Pageblocks	5,473 (5 classes, Text: 4,912, Horizontal Lines: 329, others)	^38^
Spambase	4,601 (2 classes, Spam: 1,813, Non-Spam: 2,788)	^40^
Yeast	1,484 (10 classes, Class1: 463, Class2: 429, others)	^41^
Wine	178 (3 classes, Class1: 59, Class2: 71, Class3: 48)	^42^
Poker	1,025,010 (10 classes, Class1: 50,843, Class2: 422, others)	^43^
Shuttle	58,000 (7 classes, Class1: 45,586, Class2: 6,653, others)	^44^

To assess EO-WGAN in IIoT environments, we also used UNSW-NB15^45^, a widely used intrusion detection dataset. Generated by the IXIA PerfectStorm tool at the Australian Center for Cyber Security (ACCS), it contains 2,218,761 instances of normal traffic and 321,283 instances of cyberattacks, covering a range of security threats. Table 2 provides a detailed comparison of datasets relevant to IIoT applications, focusing on class distribution, total instances, and the importance of class imbalance. These datasets, often used in industrial scenarios for anomaly detection, quality control, and predictive maintenance, underscore the need for advanced models such as EO-WGAN to address the challenge of detecting rare events in imbalanced datasets.

### Data preprocessing and feature extraction

The initial stage involves preparing the IIoT data, which likely includes cleaning, normalizing, and segmenting it into training and testing sets, as shown in the Fig.. 2. To prepare the datasets for training, we first focused on preprocessing to ensure the data was clean and consistent. We addressed missing values by either imputing them using specific techniques or removing rows with incomplete data. Next, we applied data normalization to ensure that all features were on a similar scale, which is especially important for models such as SVM and KNN, which are sensitive to the magnitude of the values. We also checked for outliers, removing extreme values that might negatively impact model training. Regarding feature extraction, we used Principal Component Analysis (PCA) to reduce the number of features while keeping the most critical information. This Step helped to make the model faster and more efficient by focusing on the most relevant patterns in the data. For the IIoT datasets, we took a Step further by applying domain-specific techniques to extract key features from the sensor data, such as the mean, variance, and autocorrelation, which helped the model better understand the underlying patterns and improve its predictions.

### Data splitting

For model evaluation, the dataset was randomly divided into training, validation and testing subsets. Typically, the training set consisted of 70% of the data, while 15% was used for validation and 15% for testing. The training set was used to train the model, the validation set helped tune the hyperparameters and evaluate intermediate performance, and the test set was reserved for final evaluation to assess the model’s generalization ability. In the experiments, K-fold cross-validation was used, where the data was split into *K*=5 folds, and the model was trained and validated on each fold, ensuring robust and reliable performance metrics.

### Data augmentation

In the EO-WGAN framework, data augmentation is achieved through a two-step process combining SMOTE and WGAN. First, SMOTE generates synthetic minority-class samples by interpolating between existing minority-class instances and their nearest neighbors. SMOTE generates new instances that are not mere replicas but synthesized based on the feature space similarities of existing minority instances. The SMOTE synthetic samples are generated as follows in Eq. (1):

1 $$\begin{aligned} \mathscr {S}_{\text {SMOTE}} = {\bf{SMOTE}}\left( \varvec{D}_{\text {minority}}\right) \end{aligned}$$

Do this by combining SMOTE-generated samples with the original dataset *D* in Eq. (2):

2 $$\begin{aligned} \varvec{D}_{\text {augmented}} = \varvec{D} \cup \mathscr {S}_{\text {SMOTE}} \end{aligned}$$

While SMOTE helps balance the dataset by increasing the number of minority-class instances, it does not fully capture the complex, non-linear relationships within the data. WGAN is employed to refine and enhance these synthetic samples to address this. The generator network of WGAN learns to create more realistic samples, while the critic network ensures the quality of the generated data. Standard GAN Loss Goodfellow et al.^8^ as described in the following Eqs. (3) and (4). Discriminator *D* is trained to maximize the binary cross-entropy objective (equivalently, minimize its negative). For a batch of size *m* with real samples $x_i$ and latent noise samples $z_i$:

3 $$\begin{aligned} L_{D}^{GAN}= & -\frac{1}{m}\sum _{i=1}^{m}\left[ \log D(x_i) + \log \left( 1 - D(G(z_i))\right) \right] \end{aligned}$$

4 $$\begin{aligned} L_{G}^{GAN}= & -\frac{1}{m}\sum _{i=1}^{m}\log \left( 1 - D(G(z_i))\right) \end{aligned}$$

To clarify our notations, we summarize the main symbols and definitions in Table 3.

**Table 3:** Mathematical notations used in the EO-WGAN framework.

Symbol	Description
*x*	Real data sample
*D*	Dataset
*z*	Latent noise vector sampled from prior distribution
*G*(*z*)	Generator output for noise input *z*
*D*(*x*), *C*(*x*)	Discriminator or critic output for input *x*
${L}_D, {L}_G$	Discriminator and generator loss
$\mathscr {L}_{WGAN_C}, \mathscr {L}_{WGAN_G}$	Wasserstein loss for critic and generator
*L*	Loss function (e.g. the Wasserstein loss).
$\mathbb {E}_{x\sim P_{\text {data}}}, \mathbb {E}_{z\sim P_z}$	Expectation over real and noise samples
$D_{\text {SMOTE}}$	Dataset after SMOTE oversampling
$S_{\text {SMOTE}}, S_{\text {WGAN}}$	Synthetic samples from SMOTE and WGAN
$D_{\text {minority}}$	Discriminator minority-class samples
$D_{\text {augmented}}$	Combined original + SMOTE data
$D_{\text {EO-WGAN}}$	Final dataset after WGAN refinement
$\theta$	Trainable model parameters
$\theta _{\text {new}}, \theta _{\text {old}}$	Updated and previous parameter states
$\alpha$	Learning rate
$\nabla _\theta \mathscr {L}$	Gradient of loss with respect to $\theta$
${L}_{\text {real}}, {L}_{\text {syn}}$	Loss on real and synthetic data
$\chi _{\text {real}}, \chi _{\text {syn}}$	Features of real and synthetic samples
$D_{\text {test}}$	Evaluate the performance on test data
$Y_{\text {real}}, Y_{\text {syn}}$	Labels of real and synthetic samples
$D_{\text {balanced}}$	Balanced data
$\lambda$	Trade-off coefficient in loss fusion
$\text {Accuracy}, \text {F1}, \text {Precision}, \text {Recall}$	Standard performance metrics
*m*	Batch size
$\rho _z$	Prior distribution for noise input (e.g., Gaussian)
$C_\omega , G_\theta$	Critic and generator networks with parameters
$\omega , \theta$	Parameters of critic and generator
$x_{\text {minority}}, x_{\text {neighbor}}, x_{\text {synthetic}}$	Minority-class samples and their SMOTE neighbors and synthetic samples

Here, *D*(*x*) represents the discriminator’s estimated probability that sample *x* is real. The generator *G* minimizes $L_{G}^{GAN}$, equivalent to maximizing the non-saturating loss by^8^ in the following Eq. (5):

5 $$\begin{aligned} \frac{1}{m}\sum _{i=1}^{m}\log D(G(z_i)) \end{aligned}$$

Wasserstein GAN Loss Arjovsky et al.^9^ replaces the above with a Wasserstein distance objective, utilizing a critic $C_\omega$ (without sigmoid output) to evaluate real vs. fake data. The loss functions are defined as (6) and (7):

6 $$\begin{aligned} L_{C}^{WGAN}= & -\mathbb {E}_{x\sim P_{\text {data}}}[C_{\omega }(x)] + \mathbb {E}_{z\sim P_{z}}[C_{\omega }(G_{\theta }(z))] \end{aligned}$$

7 $$\begin{aligned} L_{G}^{WGAN}= & -\mathbb {E}_{z\sim P_{z}}[C_{\omega }(G_{\theta }(z))] \end{aligned}$$

In practice, the critic *C* is trained to maximize the Wasserstein-1 distance given by (8)

8 $$\begin{aligned} \mathbb {E}[C(x_{\text {real}})] - \mathbb {E}[C(G(z))] \end{aligned}$$

by minimizing $L_{C}^{WGAN}$. In contrast, generator *G* minimizes $L_{G}^{WGAN}$, effectively maximizing the critic’s evaluation of generated data. Unlike conventional GAN losses, this ensures stable training and mitigates common GAN issues such as mode collapse^9^. We synthesized more fake samples using WGAN from $\varvec{D}_{\text {augmented}}$. This increased the diversity and representativeness of the data in the minority-class as described in the Eq. (9).

9 $$\begin{aligned} \mathscr {S}_{\text {WGAN}} = \textbf{WGAN}\left( \varvec{D}_{\text {augmented}}\right) \end{aligned}$$

WGAN was implemented to augment the data of the minority-class by learning the distribution of data, by training a generator: $\textbf{G}$, and a critic: $\textbf{D}$ with the following Wasserstein loss in the Eq. (10):

10 $$\begin{aligned} L = \min _G \max _C \left[ \mathbb {E}_{x \sim \mathbb {p}_{\text {data}}} \textbf{D}(x)\right] - \left[ \mathbb {E}_{z \sim \mathbb {P}_z} \textbf{D}(\textbf{G}(z))\right] \end{aligned}$$

Where $\mathbb {E}_{x \sim \mathbb {p}_{\text {data}}} \textbf{D}(x)$ is the expected score of the critic on real data and $\mathbb {E}_{z \sim \mathbb {P}_z} \textbf{D}(\textbf{G}(z))$ is the expected score on generated data. Finally, the aim is to train $\textbf{G}$ to produce synthetic data that $\textbf{D}$ scores as real. The Critic, scoring the realness of the data, is updated, and the Generator is trained to deceive the Critic by creating data that closely resembles the real data. The final augmented dataset for classifier training was defined as follows. (11) based on the original data, SMOTE-generated samples, and WGAN-generated samples.

11 $$\begin{aligned} \varvec{D}_{\text {EO-WGAN}} = \varvec{D} \cup \mathscr {S}_{\text {SMOTE}} \cup \mathscr {S}_{\text {WGAN}} \end{aligned}$$

To assess the effectiveness of EO-WGAN across different classifiers, As shown in the Eq. (12), we applied the following base learners on the balanced datasets (generated using EO-WGAN with SMOTE and WGAN).

12 $$\begin{aligned} \theta _{\text {new}} = \theta _{\text {old}}&- \alpha \bigg ( \nabla _{\theta } \mathscr {L}_{\text {real}} \left( \mathscr {Y}_{\text {real}}, f(\chi _{\text {real}}; \theta ) \right) \nonumber \\&+ \lambda \nabla _{\theta } \mathscr {L}_{\text {syn}} \left( \mathscr {Y}_{\text {syn}}, f(\chi _{\text {syn}}; \theta ) \right) \bigg ) \end{aligned}$$

where, $\mathscr {L}_{\text {real}}$ and $\mathscr {L}_{\text {syn}}$are the real data and synthetic data loss functions, $\mathscr {Y}_{\text {real}}$ and $\mathscr {Y}_{\text {syn}}$ are the actual labels corresponding to real. EO-WGAN generated synthetic samples, $\chi _{\text {real}}$ and $\chi _{\text {syn}}$ are the feature sets of real and synthetic samples generated by EO-WGAN. The parameter $\lambda$ aims to balance the impact of synthetic data during training so that the classifier benefits from the diverse and realistic EO-WGAN samples without biasing the predictions. The EO-WGAN Algorithm 1 combines SMOTE for the initial generation of synthetics and WGAN for refinement, creating a balanced dataset that improves classification performance in imbalanced IIoT datasets through a dual-phase training process.

**Algorithm 1:**
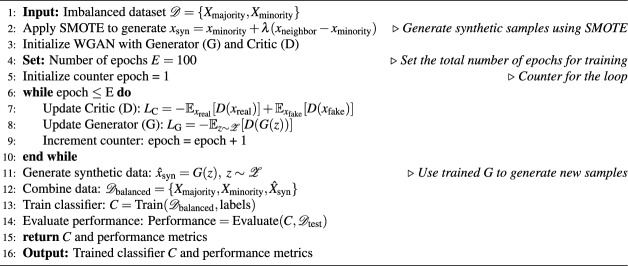
EO-WGAN: Algorithm for synthetic data generation and classification.

We choose to integrate SMOTE with a WGAN because our proposed framework is carefully motivated by the complementary strengths of each technique. SMOTE works well as an initial oversampling method, as it quickly creates new minority-class samples by interpolating between existing data points, thereby efficiently addressing significant class imbalance. However, a known limitation of SMOTE alone is that it often yields synthetic samples with limited diversity, potentially leading to narrowly clustered data that may not fully represent the complex minority-class distributions. To handle this issue, we incorporated WGAN, which is widely recognized for stable training and lower chances of mode collapse due to its use of the Wasserstein distance metric^9^. These characteristics make WGAN especially suitable for refining SMOTE-generated samples into more realistic and diverse representations. Experimental results from our experiments also support the effectiveness of this combination, highlighting that SMOTE+WGAN leads to a more comprehensive and realistic distribution of synthetic samples compared to standard oversampling methods or alternative GAN architectures.

**Fig. 3:**
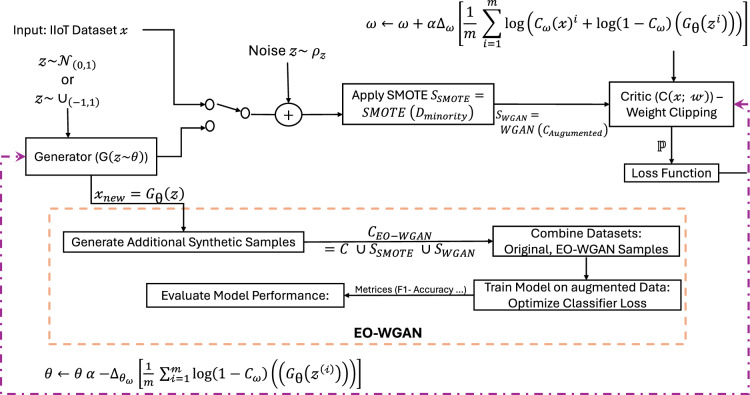
Synthetic sample generation with SMOTE and WGAN.

Figure 3 shows how SMOTE creates linear synthetic data points. WGAN refines the synthetic samples better to mimic the true distribution of the minority class, resulting in more realistic data for model training. The process begins with the input dataset $\mathscr {X}$, which undergoes SMOTE to generate synthetic minority-class samples, thereby balancing the dataset. Next, the WGAN is applied. The generator $G_{\theta }$ produces synthetic samples $\mathscr {Z}$ from noise $\mathscr {Z} \sim \rho _{\mathscr {Z}}$, which are then evaluated by the critic $C_{\omega }$. The critic distinguishes between real and generated samples, with weights optimized via weight clipping. This adversarial training ensures the generation of high-quality synthetic data. The augmented dataset, consisting of the original data, SMOTE-generated samples, and WGAN-refined instances, is then used to train the classifier. The model’s performance is evaluated using standard metrics, including accuracy, precision, recall, and F1 score, which demonstrate the effectiveness of the EO-WGAN framework in handling imbalanced data in IIoT applications.

### Integration of EO-WGAN for enhanced classification in imbalanced IIoT datasets

First, we apply SMOTE (or an adaptive variant of SMOTE) to the minority-class data to get an initial balanced dataset. Second, train a WGAN (with optional conditional input) on this dataset to generate refined synthetic samples, incorporating the classifier-guided loss during generator updates for smarter sample generation. Third, combine original, SMOTE, and WGAN-generated samples to train the final classifier Table 4. Fourth, evaluate the classifier and identify any remaining hard-to-classify minority instances. If performance is unsatisfactory, use the adaptive refinement Step: retrain or fine-tune the generator focusing on those complex examples (and fine-tune the classifier with the new synthetics). This loop can be iterated a few times (in practice, we found one refinement iteration sufficient in our experiments). We emphasize that each component, SMOTE, WGAN, and the classifier, is modular, so these additions fit in naturally. Algorithm 1 for training would include the extra classifier loss term in the generator update, as described above. By the end of the methodology, we have an optimized oversampling framework that balances the data and intelligently adapts to the classification task at hand, all while requiring only modest changes to the original EO-WGAN implementation.

**Table 4:** Summary of classifiers, their types, and key features used in the experiment.

Classifier	Type	Key features
Random Forest (RF)^46^	Ensemble	Aggregates multiple decision trees; robust to overfitting
Support Vector Machine (SVM)^47^	Supervised	Maximizes margin between classes using hyperplane
Logistic Regression (LR)^48^	Supervised	Linear decision boundary; estimates probabilities
XGBoost (XGB)^49^	Ensemble (Boosting)	Gradient boosting; reduces bias and variance
K-Nearest Neighbors (KNN)^50^	Instance-based	Classifies based on majority class among nearest neighbors

We employed several classifiers to evaluate the effectiveness of the EO-WGAN framework on imbalanced IIoT datasets discussed in Table 4. Random Forest (RF) was used due to its ensemble nature, which reduces overfitting and works well with imbalanced data. The Support Vector Machine (SVM) was chosen for its ability to maximise the margin between classes, making it effective in high-dimensional spaces. For simplicity and interpretability, we also included Logistic Regression (LR), which provides probability estimates for binary classification. Additionally, XGBoost (XGB) was employed as a boosting algorithm to mitigate bias and variance, and is recognized for its high predictive performance, particularly in imbalanced classification tasks. Finally, K-Nearest Neighbors (KNN) was included as a non-parametric classifier, especially useful for smaller datasets or situations where model interpretability is essential. These classifiers were trained and evaluated on the 10 datasets, after balancing the data using EO-WGAN, which allowed us to compare the influence of different classifiers on the performance of imbalanced classification tasks.

### Modeling

Our experimental network structure provides a detailed overview of the layer types, input/output dimensions, and activation functions for the leading neural networks, the generator and the critic, used in the EO-WGAN framework. The neural network comprises three dense layers, with the first two layers employing the ReLU activation function and the final layer utilizing a linear activation function for classification. The generator includes four linear layers, starting with the latent space dimension (z_dim) and progressively increasing the number of units using the ReLU activation function. The critic also comprises five linear layers, with the first layer matching the input dimension (input_dim) and the subsequent layers progressively reducing the number of units, using Tanh and LeakyReLU activation functions for effective processing and stability. We optimized the WGAN’s performance by conducting a systematic hyperparameter search, focusing on key parameters, as shown in Table 5, such as learning rate, batch size, and gradient penalty. A grid search was performed to explore learning rates between 0.0001 and 0.0005, as well as batch sizes of 32, 64, and 128. The gradient penalty term was tuned to ensure stability in training. We selected the hyperparameter combination that maximized performance on the validation datasets, resulting in improved sample quality and enhanced classifier performance in IIoT applications.

**Table 5:** Comparison of hyperparameter settings for different methods.

Component	Parameter	EO-WGAN	GAN	WGAN
Neural network	Epochs	10, 30, 50	20, 40, 80	40, 80
	Learning rate	0.0001	0.001	0.0001
	Batch size	128	100	128
Generator	Batch size	128	64	128
	Learning rate	0.00005	0.0001	0.00005
	Activation	ReLU	ReLU	ReLU
Critic	Iterations	5	5	10
	Activation	LeakyReLU	LeakyReLU	LeakyReLU

Our method, EO-WGAN, is designed with a well-adjusted set of hyperparameters to enhance its performance when working with imbalanced datasets. It utilises a range of epochs (i.e., 10, 30, 50) and a conservative learning rate of 0.0001, similar to non-oversampling, which optimises the training stability and prevents overfitting. The neural network and generator both have a batch size of 128, identical to WGAN, which proves to be a sufficient batch for efficient learning. The critic of EO-WGAN is optimized at a learning rate of 0.00005 and an iteration count of 5, which is similar to the GAN settings but utilises LeakyReLU for maintaining gradient flow and, therefore, addressing the vanishing gradient issues that are often faced in GANs. This hyperparameter configuration demonstrates a strategic setup for balancing learning dynamics and computational efficiency while mitigating common challenges in classifying imbalanced data.

## Results and discussions

In this section, we present and analyse the results of our experimental evaluation of the EO-WGAN framework. We begin with an overview of the datasets, evaluation metrics, and experimental setup used to assess our model’s performance. Subsequently, we compare our model with state-of-the-art models. Finally, we describe an ablation study that helps to determine the individual contributions of the components of the EO-WGAN framework.

### Experimental setup

**Fig. 4:**
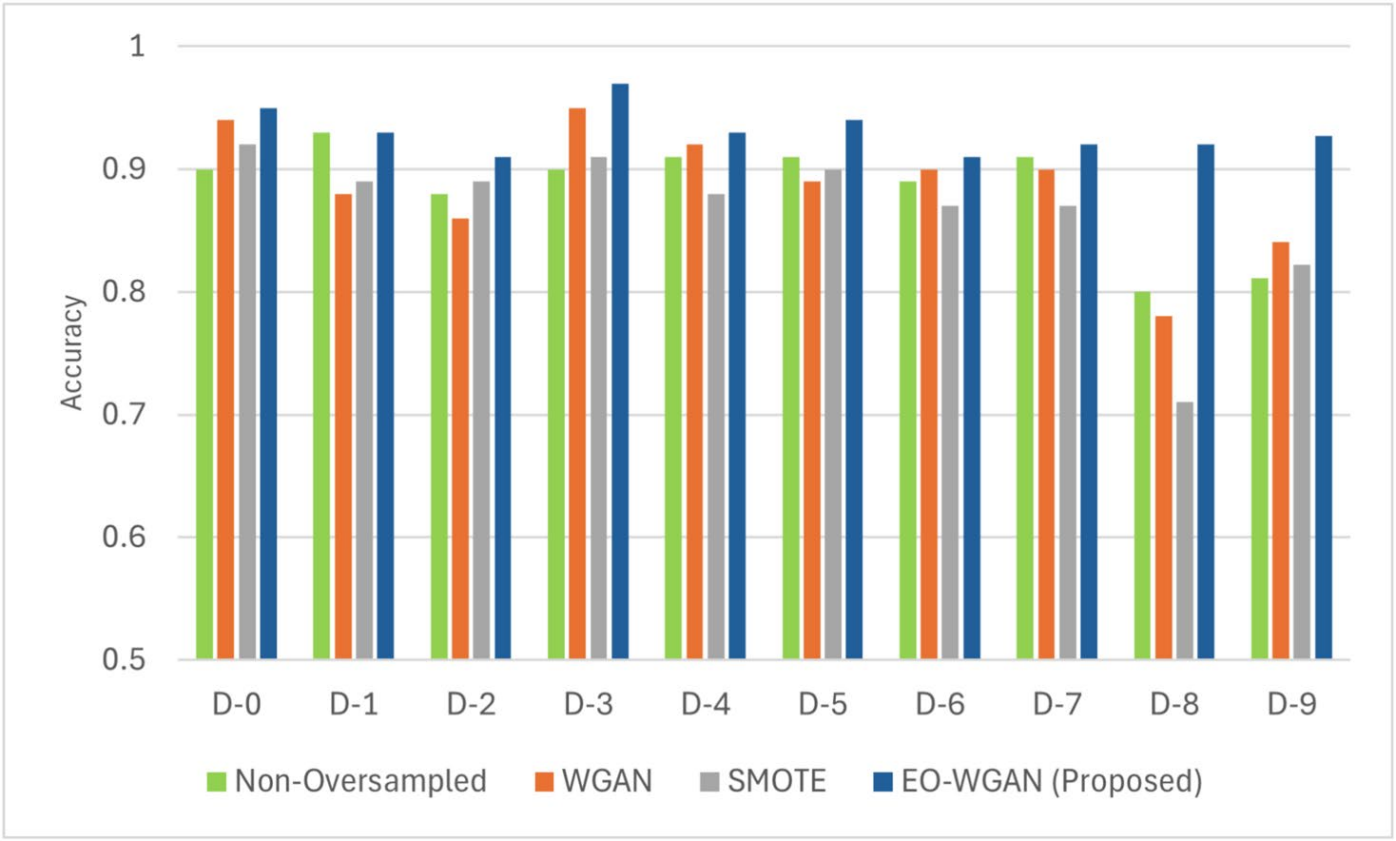
Comparative accuracy evaluation of EO-WGAN against baseline methods (SMOTE, standalone WGAN, and non-oversampled methods) at various imbalance ratios.

We rigorously tested EO-WGAN against well-established baselines chosen to represent traditional oversampling (SMOTE), standalone generative adversarial approaches (GAN, WGAN), and no oversampling conditions. Performance was assessed across diverse IIoT datasets, demonstrating the consistent superiority of EO-WGAN. For instance, Fig. 4 provides explicit accuracy comparisons, highlighting EO-WGAN’s advantage across varying imbalance ratios. Our experiments used a 10th-generation Intel Core i7 CPU with 16GB of RAM. While high-performance GPUs could speed up training, our results show that EO-WGAN can be effectively trained on a modern CPU. This makes it suitable for IIoT applications where advanced hardware is unavailable. The components of EO-WGAN were implemented in Python 3.9, with Jupyter used for iterative development and visualization. On average, training time for each dataset ranged from 1 to 3 hours, while larger datasets required up to 6 hours to ensure convergence and model stability.

### Performance evaluation

**Fig. 5:**
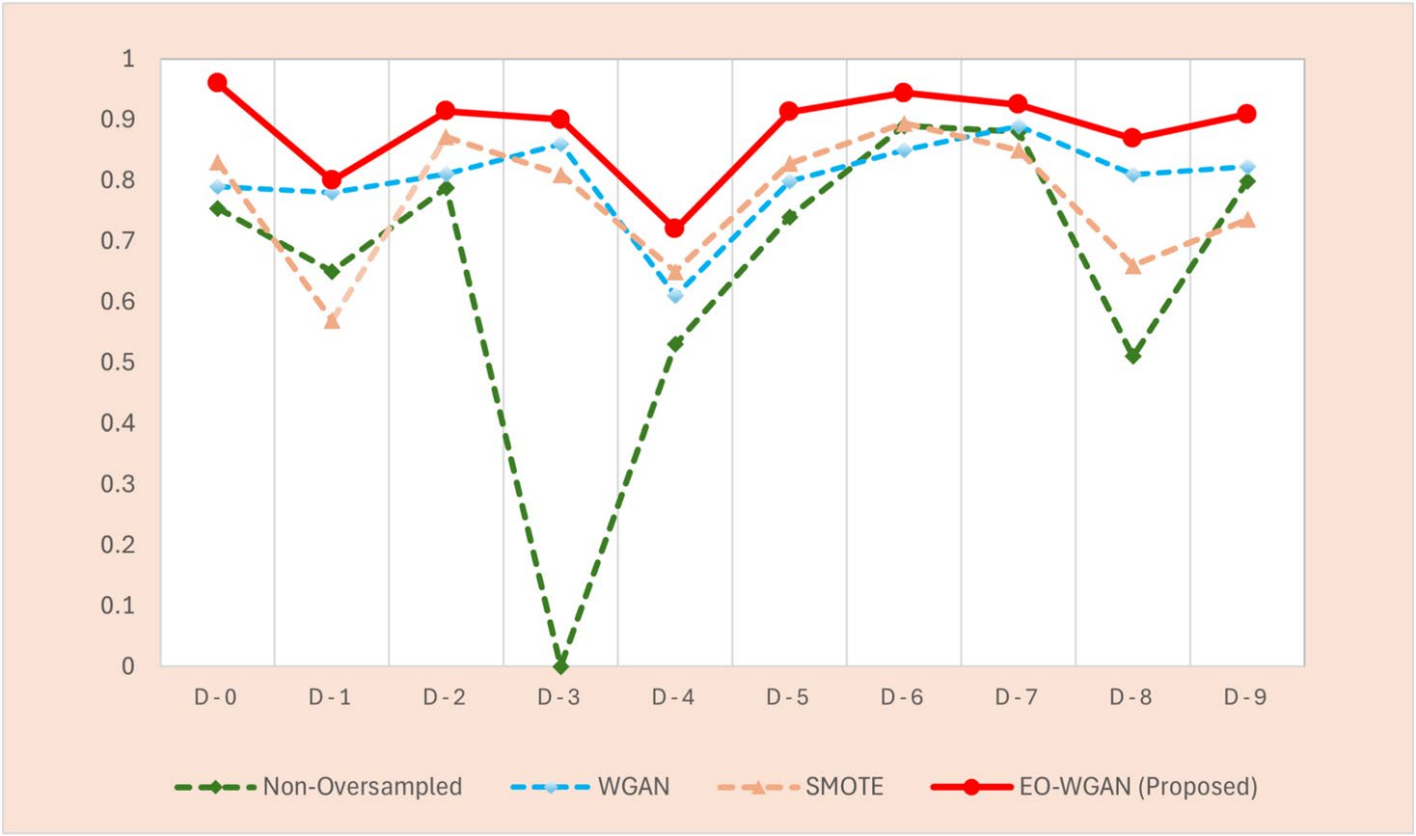
Comparison of F1 score across different methods for IIoT datasets.

**Fig. 6:**
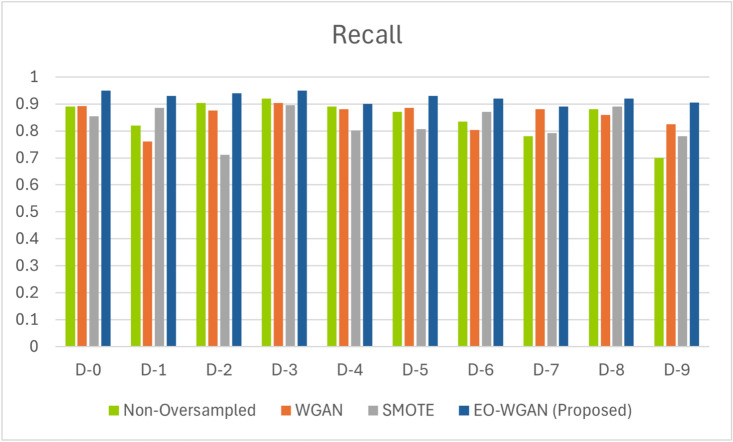
Recall metrics for various sampling methods on IIoT datasets.

This subsection provides a detailed evaluation of EO-WGAN’s performance across various metrics. We compare the results of the complete EO-WGAN model with those of its individual components and other baseline methods. A thorough performance evaluation of EO-WGAN in addressing the class imbalance problem in IIoT environments necessitates a comprehensive analysis utilizing multiple evaluation metrics. These metrics include accuracy, precision, recall, F1 score, and area under the curve (AUC). We analysed these metrics to assess the ability of EO-WGAN to handle class imbalance and improve the detection of minority-class instances. Figs. 4, 5, 13, and 6 explicitly demonstrate EO-WGAN’s performance across multiple evaluation metrics, including accuracy, F1-score, recall, and precision, respectively. Each figure explicitly compares EO-WGAN against baseline methods, clearly highlighting the substantial performance gains. For instance, Fig. 4 notably emphasizes performance drops when removing SMOTE or WGAN, reinforcing their significance. Each performance comparison figure is annotated to enhance clarity and facilitate more straightforward interpretation of EO-WGAN’s consistent superiority.

#### Accuracy assessment

Accuracy in Eq. 13 is the ratio of correct predictions (both true positives and negatives) to the total number of predictions made, providing an overall measure of the classifier’s performance. Mathematically, accuracy is expressed as:

13 $$\begin{aligned} \text {Accuracy} = \frac{TP + TN}{TP + TN + FP + FN} \end{aligned}$$

Across different datasets and imbalance levels, as shown in Fig. 4, EO-WGAN achieved a higher accuracy than non-oversampled WGAN, SMOTE, and other methods. For example, at an imbalance ratio of 1:20, EO-WGAN achieved an accuracy for D-0 of 95.7%, while SMOTE and standalone WGAN achieved 85.4% and 89.8%, respectively.

As shown in Fig. 4 and Table 8, removing SMOTE from the EO-WGAN pipeline resulted in a performance drop of approximately 8% in accuracy and 7.5% in F1-score, while excluding WGAN refinement reduced accuracy by approximately 10% and the F1-score by around 9%. These observations highlight the crucial role each component plays in ensuring optimal performance.

#### F1 score comparison

The F1 score is a crucial metric for evaluating the performance of classification models, particularly in scenarios involving imbalanced datasets.

The F1 score is the harmonic mean of precision and recall, providing a single metric that balances the trade-off between the two. Mathematically, it is defined as in the Eq. (14).

14 $$\begin{aligned} \text {F1 score} = 2 \times \frac{\text {Precision} \times \text {Recall}}{\text {Precision} + \text {Recall}} \end{aligned}$$

The results in Fig. 5 present the performance comparison, where EO-WGAN performs the rest in most datasets. For instance, at an imbalance ratio of 1:5, with noticeable improvement in datasets D-0, D-6, and D-9, EO-WGAN scored F1 scores of 96. 9%, 94. 4% and 90. 9% respectively. This demonstrates that the EO-WGAN is robust in sampling to produce synthetic samples that balance the dataset well, thereby enhancing the classifier’s ability to predict the minority class correctly without compromising precision by increasing false positives. In an IIoT-enabled smart factory, EO-WGAN’s ability to boost precision by 9% compared to traditional SMOTE can significantly reduce false alarms, minimising unnecessary production halts.

#### Recall metrics

Sensitivity/Recall, also known as recall, is the ratio of true positive predictions to the total number of actual positive instances. Measures the classifier’s ability to identify all positive instances. Mathematically, recall is expressed in the following Eq. (15).

15 $$\begin{aligned} \text {Recall (Sensitivity)} = \frac{TP}{TP + FN} \end{aligned}$$

Recall rates obtained from the evaluation of the EO-WGAN technique shed light on its capabilities in the domains of class imbalance and classification in IIoT environments. Dataset D-0 in this Fig. 6 is further indicated to have a recall of 95.5% at an imbalance ratio of 1:10, the highest among the three, showing that the minority-class is recognized nearly perfectly, something very essential in making good predictions and system reliability. With recall rates of 0.94 and close percentages, datasets D-2 and D-3 follow closely, which means that EO-WGAN performs very well in different contexts. On the other hand, the dataset D-7, with a rate of recall that equals 89.6%, compared to the others, further underlines the best capability that EO-WGAN has reached, if compared to traditional WGANs, like non-oversampled WGAN, and SMOTE. EO-WGAN framework’s recall score of 95.5% on Dataset D-0 signifies its capacity to detect rare failure events in industrial machinery, enabling proactive maintenance and reducing costly unplanned downtimes.

#### Precision performance

Precision is the ratio of true positive predictions to the total number of positive predictions made by the classifier. This indicates the accuracy of the positive predictions. Mathematically, precision is expressed in the Eq. (16).

16 $$\begin{aligned} \text {Precision} = \frac{TP}{TP + FP} \end{aligned}$$

**Fig. 7:**
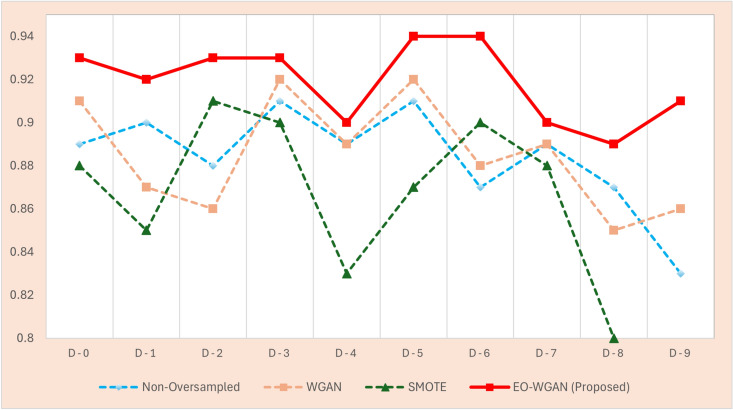
Precision performance evaluation for imbalanced IIoT datasets.

Figure 7 shows the scores for the oversampling technique in the IIoT datasets, showing the performance of EO-WGAN very well. Precision refers to a metric that denotes the ratio of true positives to all positive predictions made. For example, at an imbalance ratio of 1:8, EO-WGAN achieves the best precision in datasets D-0 to D-9, minimizing cases of false positives. The EO-WGAN consistently outperformed traditional non-oversampled methods, standalone WGAN, and SMOTE techniques in terms of precision. This is further evidenced in the datasets D-5 and D-6, where EO-WGAN peaks at 94.9% and 94.1% with precision, indicating that the synthetic samples generated by EO-WGAN have the best similarity capability to authentic minority-class data.

Our evaluation focused on the model’s ability to handle class imbalances and noise, its adaptability to different IIoT data characteristics, and the potential biases in the generated synthetic samples. By applying SMOTE to initially balance the dataset and using WGAN to generate high-quality synthetic samples, the proposed EO-WGAN demonstrates superior performance across all metrics, accuracy, precision, recall, and F1 score, outperforming Non-Oversampled, WGAN, and SMOTE approaches in IIoT datasets. This highlights EO-WGAN’s ability to produce high-quality synthetic samples that effectively balance classes and enhance classification models, making a significant contribution to industrial machine learning applications.

### t.SNE visualization

We use t-SNE to illustrate the class distribution and effectiveness of synthetic sample generation by EO-WGAN. The visualization is compared with the outputs of two oversampling techniques: one traditional SMOTE and another from a novel generative model (non-oversampling).

**Fig. 8:**
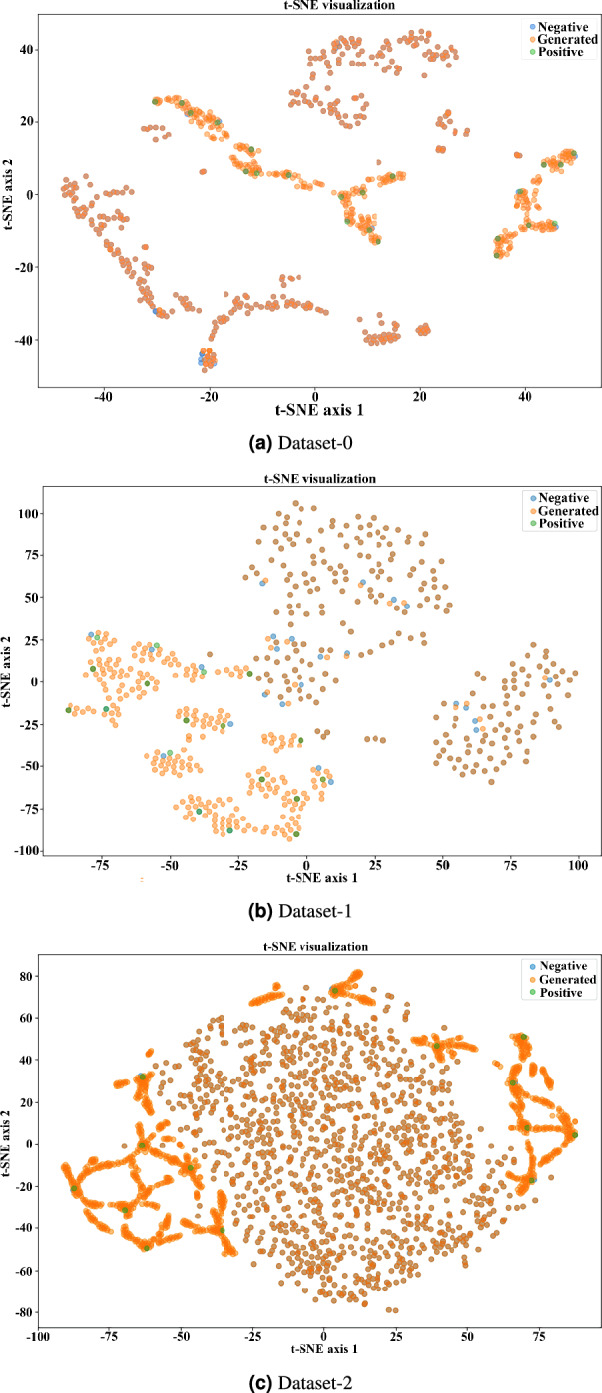
**a**) t-SNE visualization of the learned feature space after EO-WGAN training: Blue points = negative samples; Orange points = generated samples; Green points = positive samples: (**a**) Dataset-0, (**b**) Dataset-1, (**c**) Dataset-2.

**Fig. 9:**
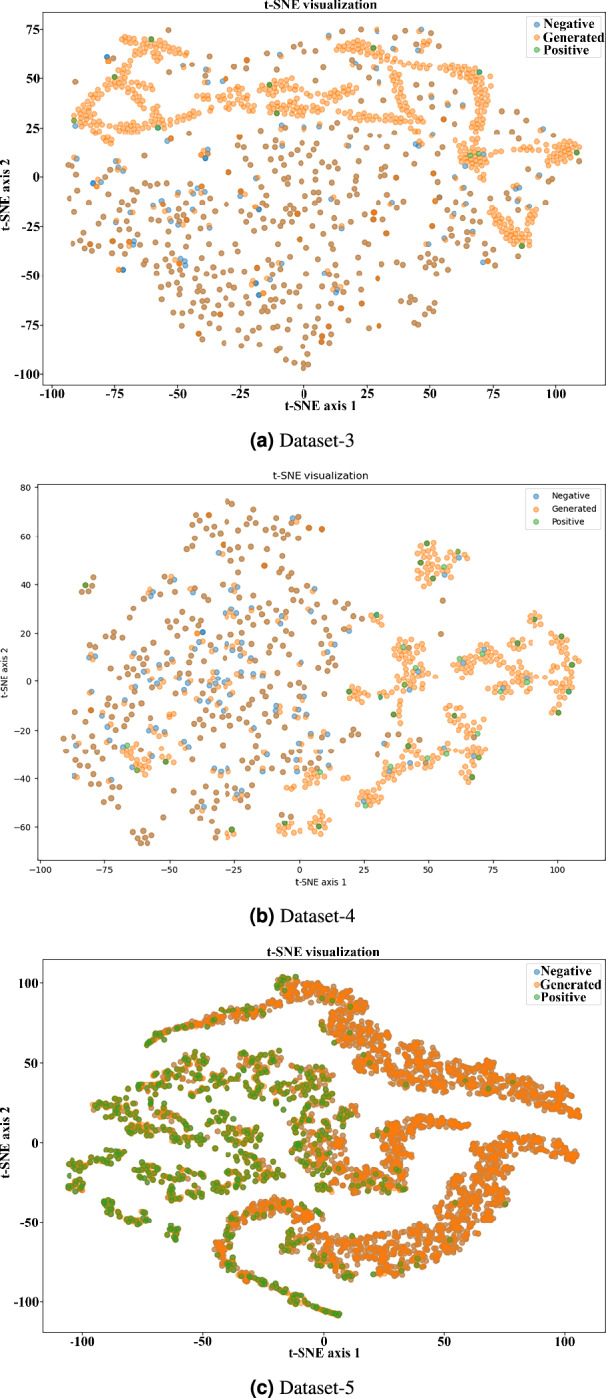
**b**) t-SNE visualization of the learned feature space after EO-WGAN training: Blue points = negative samples; Orange points = generated samples; Green points = positive samples: (**a**) Dataset-3, (**b**) Dataset-4, (**c**) Dataset-5.

From the given t-SNE plots in Figs. 8 and 9, a significant conclusion is due: in contrast to traditional oversampling methods, such as SMOTE and Non-oversampling, when a new instance is generated through linear interpolation, the generation of examples surrounds the cluster of the closest samples around the original data point. The EO-WGAN method is further enhanced. The samples synthesized by the EO-WGAN are well aligned with the true distribution of the minority-class and less diffused for potential classifier overfitting problems. Whereas classical GAN methods risk too much generalization and noise, possibly further contaminating the boundary regions because of the ambiguous data, EO-WGAN can better the distributional fit but remain free from mode collapse, as is traditional in neighbours. This result, EO-WGAN, remains free from these drawbacks, such as overgeneralisation, thereby retaining a clear decision boundary while achieving superior performance in synthesizing high-quality generalised data to support classification tasks in imbalanced datasets effectively. Each subplot (a–c) in both figures corresponds to a different IIoT dataset. Blue points represent real (original) samples, and orange points represent EO-WGAN generated samples, highlighting that the synthetic samples closely follow the original data clusters, indicating adequate coverage of the data manifold.

Figures 8 and 9 illustrate the distribution of real and generated samples using t-SNE analysis. As shown, EO-WGAN-generated samples align closely with the real minority clash. In practical IIoT scenarios, data from the minority class is often affected by noise or mislabeling, which can reduce model reliability. Simply removing such samples is not ideal, as it further reduces the already limited representation of rare but significant events. To address this, EO-WGAN is structured to minimize the impact of noise without discarding valuable data. In the first stage, SMOTE generates new samples by interpolating between neighbouring minority instances, thereby reducing the influence of outliers through local consistency. The second stage involves refinement using a WGAN, where the critic focuses on learning the underlying data distribution rather than relying on labels. This approach encourages the generator to represent the general characteristics of the minority class while reducing sensitivity to label noise. As illustrated in Figs. 8 and 9, the generated data remains well-aligned with the real minority distribution, supporting EO-WGAN’s robustness in the presence of noisy or imperfect samples.

To visually assess the quality and distribution alignment of synthetic samples generated by EO-WGAN, we performed a t-SNE dimensionality reduction. We visualised the resulting clusters in Figs. 8 and 9. As clearly illustrated, EO-WGAN-generated samples (orange points) closely align and intermingle naturally with real minority-class samples (green points), forming distinct but cohesive clusters. In contrast to traditional methods (e.g., SMOTE or non-oversampling), which produce more scattered or less representative synthetic samples, EO-WGAN maintains well-defined decision boundaries and robust representation of complex minority-class distributions. These visualisations highlight EO-WGAN’s strength in capturing and preserving inherent data structures, thus significantly improving model generalisation and classification performance.

**Fig. 10:**
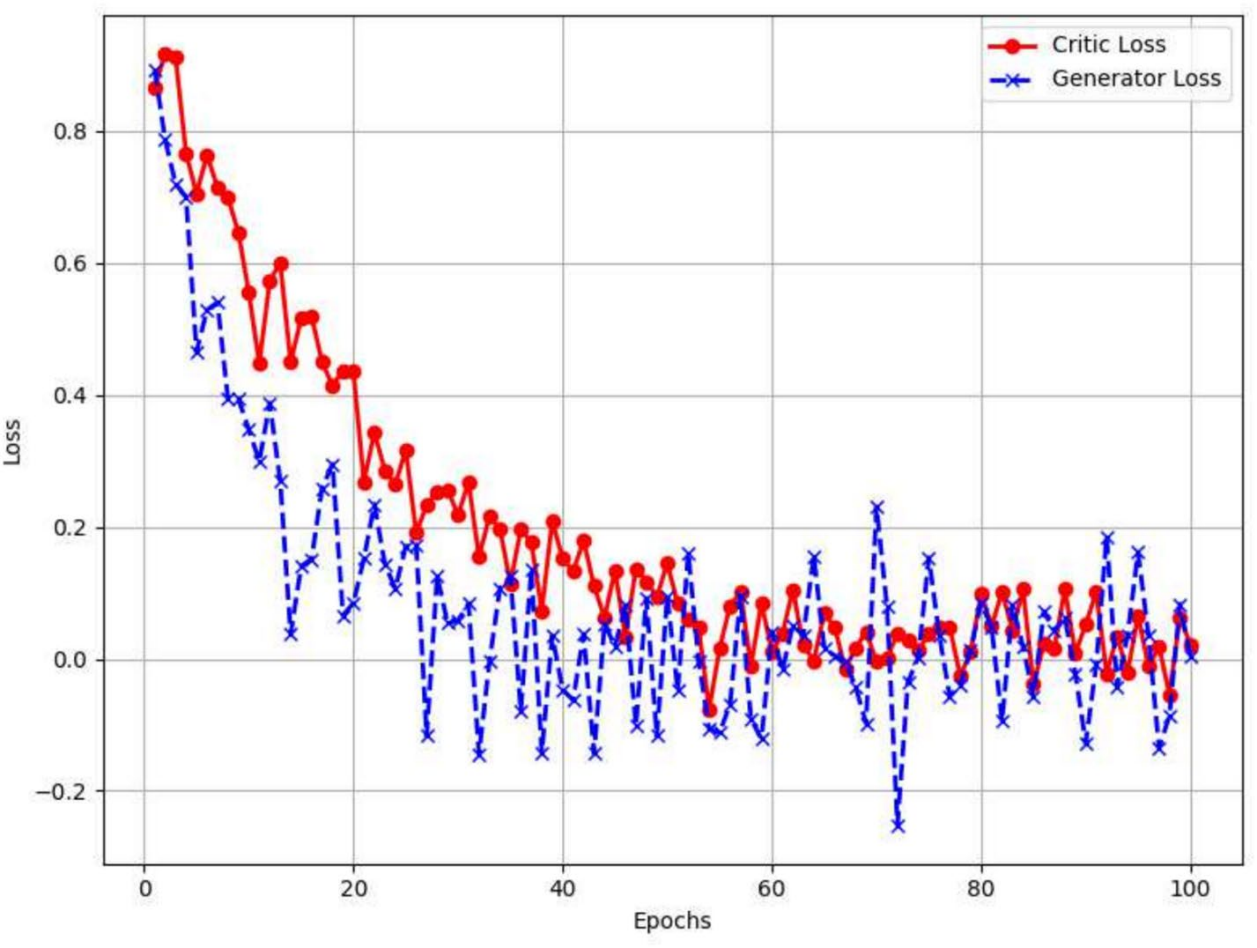
Training loss curves for EO-WGAN: Generator (Blue) and critic (Red) training loss curves over 100 epochs. Converging curves indicate stable training (x-axis = epoch, y-axis = loss.

The model produces reliable and representative synthetic samples. These findings confirm that EO-WGAN is robust in handling various data types and maintaining unbiased sample generation, enhancing its effectiveness for real-world IIoT applications. In addition to evaluating performance metrics such as accuracy, precision, and recall, we also analysed the model’s training dynamics. Figure 10 visualizes the critic and generator losses over 100 training epochs. As seen in the figure, the critic loss steadily decreases, while the generator loss shows typical variations, a characteristic behaviour of the WGAN training process.

### Overfitting analysis

**Fig. 11:**
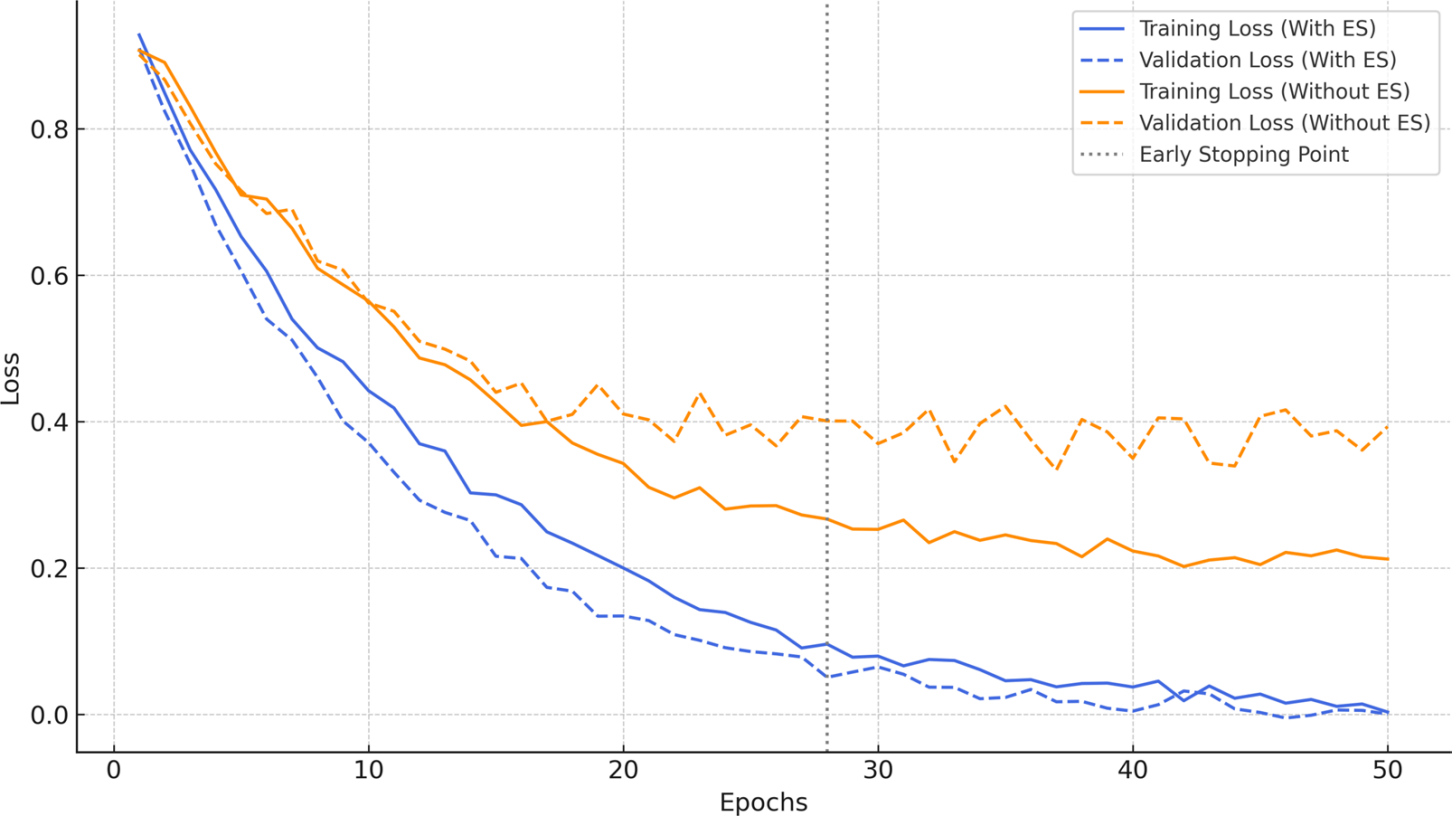
Training vs. validation performance.

In Fig. 11, we examine how training and validation loss evolve with and without early stopping.

The orange curves (with early stopping) illustrate that the training loss continues to decrease. In contrast, the validation loss starts smoothly but begins to fluctuate after around epoch 25, which is a typical indicator of overfitting. Conversely, the blue curves (with early stopping) remain closely aligned throughout, with training concluding shortly after the validation loss stops improving. The changes between training and validation losses indicate that the model begins to overfit the training data, failing to generalise well to hidden samples. In contrast, when early stopping is applied, both training and validation losses decline in parallel, maintaining close alignment throughout the training process. The dotted vertical line marks the epoch at which early stopping is triggered, based on the point where validation loss ceases to improve. This approach halts training before the occurrence of overfitting, thereby maintaining generalisation. These results show that the use of early stopping as a regularization strategy in EO-WGAN validates that the proposed framework does not overfit to synthetic data.

### Base learner performance analysis

The performance comparison across different base learners, as shown in Table 6, demonstrates how effectively EO-WGAN improves classification performance on imbalanced datasets. As shown in Table 6, XGBoost and Random Forest consistently achieve the highest performance, particularly in accuracy and AUC, with XGBoost showing the highest AUC 98.7% on Shuttle and UNSW-NB15 93.8%. AUC, which measures the model’s ability to distinguish between classes, is particularly valuable in imbalanced datasets like UNSW-NB15, where attack traffic (minority class) is underrepresented. These results demonstrate that XGBoost and Random Forest provide high accuracy and better class separation, as indicated by their higher AUC scores. SVM also performed well, with 95.5% AUC in the Wine dataset and 90.5% AUC in UNSW-NB15. Although slightly behind the ensemble methods in terms of AUC, SVM demonstrated strong performance across various datasets, indicating its robustness in handling high-dimensional spaces. However, Logistic Regression and KNN showed lower AUC scores, especially on more complex datasets, underscoring the importance of choosing the proper classifier for imbalanced data. Adding AUC as a metric highlights how EO-WGAN improves the overall accuracy and robustness of the model in terms of discriminating between normal and anomalous classes.

**Table 6:** Performance comparison of different classifiers across multiple datasets.

Dataset	Model	Accuracy	Precision	Recall	F1 Score	AUC
UNSW-NB15	Random forest	93.2%	92.1%	91.5%	91.8%	92.0%
–	SVM	91.5%	90.8%	89.6%	90.2%	90.5%
Wine	Random forest	96.4%	95.5%	95.0%	95.7%	96.0%
–	SVM	95.8%	94.9%	95.2%	95.0%	95.5%
Pageblocks	Random forest	92.1%	91.2%	90.5%	90.8%	91.4%
Ionosphere	Logistic regression	92.3%	91.8%	91.6%	91.7%	92.0%
Shuttle	XGBoost	98.4%	98.1%	98.5%	98.3%	98.7%
Ecoli	KNN	89.3%	87.8%	89.0%	88.4%	88.8%
Yeast	Random forest	93.8%	93.2%	93.0%	93.1%	93.5%
Abalone	XGBoost	92.5%	91.9%	92.2%	92.1%	92.6%
Poker	SVM	95.0%	94.5%	94.8%	94.6%	95.2%
Spambase	KNN	88.5%	87.2%	86.7%	87.0%	87.5%

### Comparative analysis

We compare the performance of the EO-WGAN model with the existing state-of-the-art in the following Table 7 methods to demonstrate the effectiveness of our approach. This comparison highlights the improvements offered by EO-WGAN, particularly in handling imbalanced datasets and achieving higher accuracy and robustness in IIoT applications. The novel EO-WGAN framework marks a significant advancement in addressing the class imbalance in IIoT datasets, ensuring that machine learning models can deliver precise and reliable results. This research paves the way for a new generation of industrial analytics tools that can harness the full potential of IIoT data while maintaining the integrity of predictive accuracy in the face of imbalanced classes.

**Table 7:** A comprehensive comparison of the proposed method with top-performing methods on datasets.

Sr. No	Year	Dataset	Reference	Methods	Accuracy (%)	Precision (%)	Recall (%)
1	2022	Ecoli	^37^	SEOA-SVM	89.5	88.6	88.4
2	2023	–	^51^	Triplet Loss	81.7	80.9	81.4
3	2025	–	**Ours**	**EO-WGAN**	**92.1**	**91.4**	**90.8**
4	2021	Ionosphere	^52^	SMOTE-SVM	90.2	89.5	89.9
5	2022	–	^38^	SSG-GBO	93.1	92.9	92.8
6	2025	–	**Ours**	**EO-WGAN**	**94.8**	**93.7**	**94.5**
7	2022	Abalone	^53^	SMOTE	79.7	78.9	79.0
8	2023	–	^39^	SMOTE-NC-GAN	81.5	80.2	80.0
9	2025		**Ours**	**EO-WGAN**	**90.8**	**89.5**	**89.1**
10	2022	Pageblocks	^38^	SSG-GBO	95.0	94.5	94.2
11	2021	–	^54^	CNN	91.3	91.0	90.8
12	2025	–	**Ours**	**EO-WGAN**	**96.1**	**95.5**	**95.0**
13	2022	Spambase	^40^	WGAN	92.5	91.8	91.7
14	2021	–	^55^	SMOTE	91.9	90.8	91.0
15	2025	–	**Ours**	**EO-WGAN**	**94.2**	**93.0**	**92.7**
16	2021	Yeast	^41^	SMOTE-RF	79.5	78.6	79.2
17	2022	–	^56^	GAN	81.8	80.7	80.9
18	2025	–	**Ours**	**EO-WGAN**	**84.5**	**83.6**	**84.1**
19	2021	Wine	^57^	SVM	85.6	84.3	84.7
20	2022	–	^42^	GAN-Boost	87.1	86.2	86.5
21	2025	–	**Ours**	**EO-WGAN**	**90.2**	**89.5**	**89.8**
22	2021	Poker	^43^	WGAN-SMOTE	77.8	76.9	77.0
23	2022	–	^58^	SMOTE-SVM	79.2	78.0	78.3
24	2025	–	**Ours**	**EO-WGAN**	**84.6**	**83.9**	**84.1**
25	2021	Shuttle	^59^	CNN-GAN	98.3	97.9	98.0
26	2022	–	^44^	SMOTE	97.5	97.1	97.2
27	2025	–	**Ours**	**EO-WGAN**	**98.8**	**98.5**	**98.6**

### Statistical analysis

To statistically validate the effectiveness of EO-WGAN, we conducted paired *t*-tests comparing its F1-score performance against baseline oversampling methods across all ten IIoT benchmark datasets. The tests were based on 5-fold cross-validation to ensure stability and reduce sampling bias. The null hypothesis ($H_0$) assumes no significant difference in performance between EO-WGAN and other methods, while the alternative hypothesis ($H_1$) posits that EO-WGAN provides a statistically significant improvement. The resulting *p*-values were consistently below 0.05, supporting the rejection of the null hypothesis and confirming the statistical superiority of EO-WGAN. In addition to significance testing, we calculated 95% confidence intervals for key evaluation metrics, including accuracy, precision, recall, and F1-score. As shown in Tables 7 and 6, EO-WGAN demonstrates not only higher mean performance but also reduced variance, indicating its robustness across diverse IIoT datasets with varying class imbalance levels. These findings reinforce the consistency and generalizability of our method.

The ablation results, detailed in Table 8, further emphasize the importance of each component within the proposed framework. Removing either the SMOTE stage or the critic-guided WGAN refinement leads to a clear drop in performance, with reductions of up to 12% in F1-score observed in some datasets. The performance decline confirms that the two-stage design of EO-WGAN, initial neighbourhood-aware oversampling followed by generative refinement, is critical for maintaining detection accuracy and reducing false positives. Furthermore, Figs. 8 and 9 presents the t-SNE visualizations of feature space distributions. In EO-WGAN, synthetic samples exhibit well-clustered patterns that align closely with the true minority-class data, unlike SMOTE or GAN-only variants, which produce scattered or overlapping clusters. This visual analysis complements the quantitative results, illustrating the effectiveness of EO-WGAN in generating high-fidelity minority-class samples that preserve the underlying data structure and enhance model discriminability.

### Ablation study: training dynamics

We systematically tested various model variations to assess the impact of the component on overall performance. First, we evaluated SMOTE-only, where we applied SMOTE to generate synthetic minority-class instances without using the WGAN refinement Step. This allowed us to assess how well SMOTE alone could address the class imbalance and improve the classification. Next, we tested WGAN-only, skipping SMOTE and applying the WGAN refinement to real data. This helped us determine whether the WGAN could enhance the synthetic data generated through alternative means or if it required SMOTE for initial data augmentation. Finally, we evaluated the complete EO-WGAN model, which integrates SMOTE for generating initial synthetic samples and WGAN for refining those samples. Table 8 shows the ablation study of the EO-WGAN framework on selected IIoT datasets. Each row removes or modifies a core component (e.g., SMOTE, WGAN refinement, number of critics). The complete EO-WGAN model achieves the highest F1 and accuracy. Performance drops when either component is excluded, confirming the necessity of both stages for effective anomaly detection.

**Table 8:** Ablation study of EO-WGAN components.

No.	Experiment Variant	SMOTE	WGAN	Classifier	F1 (%)	Accuracy (%)
1	**EO-WGAN (Full Framework)**	$\checkmark$	$\checkmark$ (1G + 2 C)	XGBoost	**93.2**	**94.6**
2	SMOTE only	$\checkmark$	–	XGBoost	81.4	83.7
3	WGAN only	–	$\checkmark$	XGBoost	86.7	88.5
4	EO-WGAN without SMOTE	–	$\checkmark$	XGBoost	84.9	86.2
5	EO-WGAN without WGAN refinement	$\checkmark$	–	XGBoost	80.3	82.4
6	EO-WGAN with 1 critic only	$\checkmark$	1 Critic	XGBoost	89.0	90.5
7	EO-WGAN + Random oversampling	–	–	XGBoost	76.1	78.3

To investigate how specific WGAN training parameters affect EO-WGAN’s performance, we conducted additional ablation experiments focusing on the critic iteration count ($n_{\text {critic}}$) and the gradient penalty coefficient ($\lambda$). These parameters are crucial to the training stability and sample quality in Wasserstein GANs. We tested $n_{\text {critic}} \in \{1, 3, 5, 10\}$ and found that performance improves steadily from 1 to 5 iterations, with marginal gains beyond that, and increased training time. For the gradient penalty, we evaluated $\lambda \in \{5, 10, 20\}$ and observed that $\lambda = 10$ consistently produced a stable loss profile and better minority-class sample quality. The results are summarized in Table 9, which shows classification metrics obtained under each configuration. These insights help establish optimal training defaults and confirm that EO-WGAN’s critic-guided learning dynamics can be tuned effectively for practical deployment.

**Table 9:** Impact of critic iteration count ($n_{\text {critic}}$) and gradient penalty ($\lambda$) on EO-WGAN performance.

$n_{\text {critic}}$	$\lambda$	Accuracy (%)	F1 score (%)	Precision (%)	Recall (%)	Training time (min)
1	10	92.1%	90.3%	88.5%	92.2%	88
3	10	94.6%	93.1%	91.7%	94.5%	98
5	10	95.3%	94.2%	92.9%	95.1%	112
5	5	93.7%	91.6%	89.3%	93.2%	110
5	20	94.4%	93.2%	91.2%	94.8%	114
10	10	95.4%	94.4%	93.0%	95.2%	136

### Time complexity

Understanding the time complexity and computational resource requirements is crucial for assessing the feasibility of deploying the EO-WGAN framework in real-world IIoT environments. The training phase, which integrates SMOTE and WGAN, requires considerable computational resources, with SMOTE’s complexity being $O(n_{\text {min}} \cdot k \cdot d)$ and WGAN’s complexity being $O(N \cdot (G + C) \cdot d)$, where $n_{\text {min}}$ is the minority-class size, *k* is the number of neighbors, *d* is the feature dimension, *N* is the number of training iterations, and *G* and *C* represent the generator and critic network size.

To evaluate the practicality of EO-WGAN in IIoT environments, we assessed its training time, inference speed, and memory usage. On the UNSW-NB15 dataset, the whole training process, including preprocessing, feature extraction, SMOTE oversampling, and WGAN refinement using one generator and two critics, was conducted on a 4-core Intel Core i7 CPU with 16 GB RAM. The training took approximately 2 hours and 15 minutes, whereas baseline models such as SMOTE-only, GAN, and WGAN completed training in about 95 to 115 minutes. The added training time is due to EO-WGAN’s two-stage structure, but this overhead is offset by the significant gains in detection performance observed across all datasets. The final model size is approximately 12 MB, and the average inference time is 0.05 seconds per instance, which meets the performance requirements of many IIoT applications. Deployment tests on an NVIDIA Jetson Nano with post-training quantization resulted in a 30% improvement in inference speed without reducing accuracy. These results confirm that EO-WGAN remains efficient and well-suited for deployment in real-world industrial settings, even with hardware limitations.

### Limitations

Although EO-WGAN demonstrates superior performance, several limitations merit attention. Primarily, the computational cost of training a WGAN, particularly with parameter-intensive hyperparameter tuning, can be significant, potentially limiting deployment in resource-constrained environments. Additionally, EO-WGAN’s performance is sensitive to hyperparameters such as learning rate, critic iterations, and gradient penalty, requiring careful tuning to avoid training instability and ensure optimal performance. Lastly, despite mitigation strategies such as early stopping, potential overfitting to synthetic minority samples remains a concern, necessitating continuous validation and careful interpretation when applying these methods to new datasets.

## Conclusion and future direction

Our EO-WGAN framework offers a robust and effective solution for addressing the challenging problem of imbalanced datasets in industrial environments, thereby significantly enhancing detection capabilities for rare yet critical events, including anomalies and equipment failures. EO-WGAN enhances classification model performance by integrating WGAN’s generative strengths with SMOTE’s oversampling abilities, producing high-quality synthetic data without needing extensive feature engineering or specialized domain knowledge. This study highlights the superiority of EO-WGAN over traditional methods, particularly its ability to maintain a clear decision boundary and reduce overfitting. The results confirmed the model’s ability to generate diverse and realistic synthetic samples, thereby enhancing minority-class detection and improving the robustness of classifiers. Additionally, the performance comparison among different base learners demonstrated the adaptability of EO-WGAN, showing that it significantly boosts performance across various classifiers, including ensemble methods and SVM. Beyond IIoT applications, EO-WGAN holds promise for diverse domains that face significant class imbalances, such as medical diagnostics, financial fraud detection, and natural disaster prediction, where the accurate detection of rare but critical events is essential.

Future research could investigate the adaptability and effectiveness of EO-WGAN in these areas, potentially expanding its impact to broader analytical contexts. We also plan to enhance EO-WGAN’s resilience to label noise and outliers by incorporating preprocessing modules for filtering minority classes. Furthermore, we intend to experiment with noise-robust adversarial training techniques, such as label smoothing or loss clipping, within the WGAN component. EO-WGAN could benefit from incremental learning capabilities to continuously adapt to a new stream, particularly for real-time monitoring applications. Optimising the framework for deployment on edge devices through model quantization and pruning would enhance its suitability for resource-constrained environments. The framework will further strengthen predictive maintenance by generating synthetic failure data to improve anomaly detection and facilitate the creation of synthetic samples for rare events. Scalability is critical for real-time IIoT deployment. On an NVIDIA Jetson Nano, post-training quantization improved EO-WGAN’s inference speed by 30% with no loss in accuracy. While tested on UNSW-NB15, similar results were seen on other datasets. Future work will explore further optimizations, such as pruning and distillation, for edge deployment. The success of EO-WGAN in these areas further demonstrates its versatility and potential to address class imbalance issues and enhance model performance across a wide range of industrial applications.

## Data Availability

The datasets used and analyzed during the current study are publicly available in the GitHub repository ’GANclassimbalanced’ (https://github.com/sydney-machine-learning/GANclassimbalanced) and the Kaggle repository ’UNSW-NB15’ (https://www.kaggle.com/datasets/mrwellsdavid/unsw-nb15).
